# Camera-Trap Assessment of Terrestrial Mammals and Ground-Dwelling Birds in the Zhangjiajie Chinese Giant Salamander National Nature Reserve, China

**DOI:** 10.3390/ani16121935

**Published:** 2026-06-22

**Authors:** Chenbo Huang, Ying Wei, Zhiyong Deng, Cheng Wang, Pengchen Zhou, Xinyu Cui, Bin Wang, Xiaoyang Mo

**Affiliations:** 1 A Key Laboratory of Biodiversity Research and Utilization, Hunan Normal University, Changsha 410081, China; hcblaile@hunnu.edu.cn (C.H.); zhoupengchen0106@163.com (P.Z.); 202510140409@hunnu.edu.cn (X.C.); 2 Affairs Center of Hunan Zhangjiajie Chinese Giant Salamander National Nature Reserve, Zhangjiajie 427400, China; 18685100701@163.com (Y.W.); dnzxdengzhiyong@163.com (Z.D.); 19118629977@163.com (C.W.)

**Keywords:** subtropical forest, Wuling Mountains, relative detection frequency, naive site occupancy, diel activity, seasonal overlap

## Abstract

Baseline information on terrestrial wildlife communities and their activity patterns is essential for protected-area management, but such information remains limited for Hunan Zhangjiajie Giant Salamander National Nature Reserve, where conservation attention has historically focused on the Chinese giant salamander and associated aquatic ecosystems. From March 2024 to August 2025, we conducted a camera-trap survey in broad-leaved and coniferous forest habitats of the reserve to document terrestrial mammals and ground-dwelling birds, evaluate taxonomic completeness, and describe diel and seasonal activity patterns. Across 43 camera-trap stations and 16,314 effective camera-trap days, we recorded 59 wildlife species, including 18 mammals and 41 ground-dwelling birds. The assemblage included nationally protected, threatened, and Chinese endemic species, indicating that the reserve’s forest habitats support important terrestrial biodiversity in addition to its aquatic conservation target. Taxonomic completeness curves suggested that the current survey captured most camera-detectable mammal and ground-dwelling bird taxa under the present sampling design, although the results should not be interpreted as a complete inventory of the reserve’s total vertebrate diversity. Annual diel activity analysis of 11 focal species showed clear temporal differentiation among ecological groups: small and medium-sized carnivores were mainly nocturnal, ground-dwelling birds, and red-hipped squirrel were primarily diurnal, and ungulates showed mixed or crepuscular-to-nocturnal tendencies. Seasonal analyses based on bioclimatic periods showed interspecific differences in activity-density distributions between the cool-dry and warm-wet seasons. However, peak-shift reliability analysis indicated that most focal species retained broadly similar main activity peaks across seasons; masked palm civet was the only species showing reliable seasonal displacement of its main activity peak. Pairwise temporal overlap analyses described temporal co-occurrence patterns among selected sympatric species but should not be interpreted as evidence of direct interaction or niche differentiation. Overall, this study provides baseline data on camera-detected terrestrial vertebrates in the reserve and supports long-term monitoring, forest habitat management, and disturbance control for terrestrial mammals and ground-dwelling birds.

## Simple Summary

Nature reserves often protect more species than their main conservation target, but this can only be confirmed through field monitoring. The Zhangjiajie Chinese Giant Salamander National Nature Reserve was established mainly to protect the Chinese giant salamander (*Andrias davidianus*) and its aquatic ecosystem, yet its forest habitats may also support important terrestrial wildlife. From March 2024 to August 2025, we used camera traps in broad-leaved and coniferous forests of the reserve to record terrestrial mammals and ground-dwelling birds. We identified 59 wildlife species, including 18 mammals and 41 ground-dwelling birds, several of which are nationally protected, threatened, or endemic to China. Mammals such as small and medium-sized carnivores were mainly active at night, while most ground-dwelling birds and squirrels were active during the day. Seasonal comparisons showed that most focal species retained broadly similar main activity times between the cool-dry and warm-wet seasons, although the masked palm civet (*Paguma larvata*) showed a reliable seasonal shift in its main activity peak. These findings show that the reserve’s forest habitats are important for terrestrial wildlife and provide useful baseline information for long-term monitoring, forest habitat management, and reducing human disturbance during sensitive activity periods.

## 1. Introduction

Reliable information on which species occur in a protected area and how they use its habitats is essential for effective wildlife conservation. Protected areas are expected to safeguard terrestrial vertebrate communities, yet their conservation outcomes are shaped by local habitat conditions, management intensity, and human pressure [1,2,3]. Global analyses have shown that reserves can support higher biodiversity than unprotected landscapes, but broad-scale camera-trap syntheses also reveal pronounced variation among protected areas in mammal assemblages, detection frequencies, and activity patterns [1,4]. These findings highlight the need for reserve-level monitoring, as without local data, it is difficult to assess the actual biodiversity value of a protected area or to design management actions that align with its ecological context.

Camera traps offer a practical way to obtain reserve-level biodiversity data, especially in mountainous forest landscapes where direct observation is often difficult. Because they can operate continuously with minimal observer disturbance, they are well-suited for recording terrestrial mammals and ground-dwelling birds, including species that are elusive, nocturnal, or sensitive to human presence [5,6]. Beyond documenting species occurrence, camera-trap records also provide time-stamped detections, making them useful for describing diel activity patterns of different species within monitored habitats [5,7]. For reserves with limited baseline information, this approach provides a standardised basis for characterising camera-detectable terrestrial vertebrate communities and their temporal activity patterns.

In northwestern Hunan, the Wuling Mountains provide an important regional context for reserve-level biodiversity monitoring. This mountainous region is characterised by complex terrain and extensive forest habitats and is known to support diverse assemblages of mammals and birds, including endemic and nationally protected species [8]. Camera-trap studies in nearby protected areas have begun to build a regional evidence base. Surveys in Gaowangjie National Nature Reserve and Badagongshan National Nature Reserve, for example, have documented mammal and bird diversity, relative detection frequencies, and diel activity patterns [9,10,11]. However, this progress has not extended evenly across all protected areas in the region. For Hunan Zhangjiajie Giant Salamander (*Andrias davidianus*) National Nature Reserve, comparable camera-trap data on terrestrial mammals and ground-dwelling birds remain scarce, leaving an important local gap in understanding the terrestrial vertebrate community using its broad-leaved and coniferous forest habitats.

Hunan Zhangjiajie Giant Salamander National Nature Reserve, established in 1995, was designated primarily to protect the Chinese giant salamander and its associated aquatic ecosystem. Previous studies have examined several aspects of the reserve, including functional zoning, habitat quality change, salamander population dynamics, fish community structure, and avian resources [12,13,14,15,16]. For example, a recent multi-source assessment updated the reserve’s bird checklist and recorded 309 bird species [15]. In contrast, the mammal fauna of the reserve remains insufficiently documented. To our knowledge, no comprehensive checklist of wild mammal species has yet been published for this protected area, and information on medium- and large-sized mammals, cryptic understory species, and the diel activity patterns of ground-dwelling birds remains limited. This gap is important because, although the reserve was established around an aquatic flagship species, its broad-leaved and coniferous forest habitats may also support important terrestrial vertebrate communities. In forested mountain reserves, aquatic systems, forest habitats, and adjacent terrestrial communities can be linked through animal movement, nutrient transfer, prey subsidies, and seasonal habitat use [17,18,19]. Incorporating terrestrial mammals and ground-dwelling birds into biodiversity assessment can therefore provide a more complete understanding of the reserve’s ecological integrity and support integrated, multi-taxon conservation management.

Beyond documenting which species are present, understanding when animals are active can provide important insight into how they use time within protected areas. Diel activity patterns may be associated with food availability, predation risk, interspecific encounters, human disturbance, and seasonal environmental variation [7]. For terrestrial mammals and ground-dwelling birds in forested protected areas, differences in activity time may reflect distinct ecological strategies and different sensitivities to environmental pressures. Examining these patterns, therefore, complements species inventories by showing not only which species occur in the reserve, but also how camera-detected species partition or overlap in their use of the daily cycle within monitored habitats. This perspective is useful for interpreting the temporal organisation of the community and for strengthening multi-taxon conservation management.

Building on this context, we used camera-trap data collected from March 2024 to August 2025 to investigate terrestrial mammals and ground-dwelling birds in the Hunan Zhangjiajie Giant Salamander National Nature Reserve. Our aim was to establish baseline information on the reserve’s camera-detected terrestrial vertebrate community and to examine the temporal activity patterns of focal species within monitored broad-leaved and coniferous forest habitats. Specifically, we asked: (1) Which terrestrial mammal and ground-dwelling bird species were detected in the reserve, and how did their relative detection frequencies and station-level occurrence vary? (2) Did the current survey effort provide an adequate basis for characterising the camera-detectable taxonomic diversity of these two groups? and (3) How did diel activity patterns differ among focal species and between the cool-dry and warm-wet seasons? We expected mammals, especially carnivores, to show stronger nocturnal activity than ground-dwelling birds, possibly reflecting differences in foraging strategy, disturbance sensitivity, and daytime exposure risk [20]. We also expected seasonal differences in activity-density distributions and peak timing to vary among focal species, because species may differ in their responses to seasonal changes in resource availability, habitat use, and environmental conditions [7]. By addressing these questions, this study provides baseline data for long-term monitoring and supports a broader, multi-taxon approach to conservation management in the reserve.

## 2. Materials and Methods

### 2.1. Study Area

**Figure 1:**
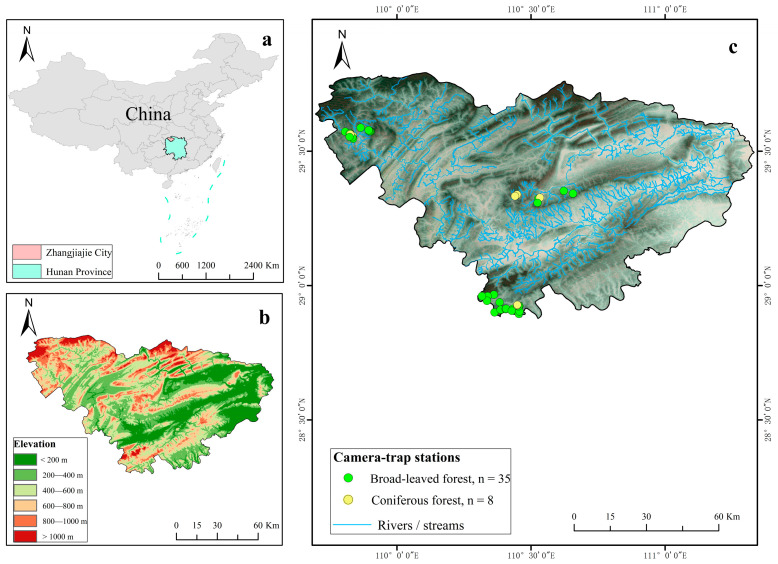
Study area and camera-trap deployment in the Hunan Zhangjiajie Giant Salamander National Nature Reserve, northwestern Hunan Province, China. (**a**) Location of Zhangjiajie City and Hunan Province within China. (**b**) Elevation gradient of the reserve. (**c**) Distribution of camera-trap stations and river systems within the reserve. Camera-trap stations were classified according to the dominant land-cover type at each location using the 2020 global 30 m fine land-cover product. Of the 43 stations, 35 were located in broad-leaved forest and 8 in coniferous forest.

**Figure 2:**
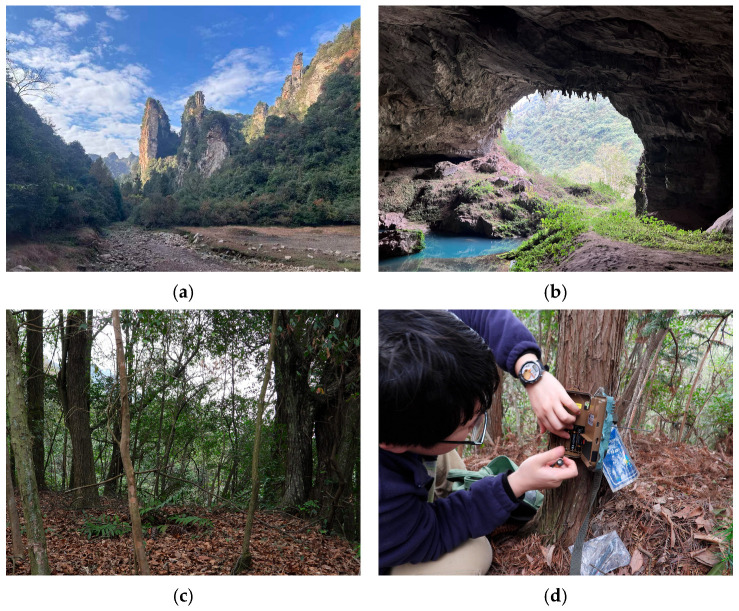
Representative habitats and camera-trap deployment in the Hunan Zhangjiajie Giant Salamander National Nature Reserve, China. (**a**) Karst gorge and stream-valley landscape in the reserve; (**b**) karst cave and riparian habitat associated with the Chinese giant salamander system; (**c**) forest understory habitat where camera traps were deployed; (**d**) installation of an infrared camera trap during field surveys.

The study was conducted in the Hunan Zhangjiajie Giant Salamander National Nature Reserve, northwestern Hunan Province, China (Figure 1a). The reserve lies in the eastern Wuling Mountains and covers 14,285 ha [21]. Elevation ranges from 58 to 1890.4 m a.s.l. (Figure 1b). The area has a humid mid-subtropical monsoon climate, with a mean annual temperature of approximately 16 °C, and is characterised by mountain forests, stream valleys, riparian habitats, and karst-related landforms. Forest cover reaches 74.75%, and the regional vegetation mainly includes mid-subtropical evergreen–deciduous broad-leaved mixed forest, evergreen broad-leaved forest, and montane dwarf forest [22,23] (Figure 2). Land use is dominated by forest land and stream-associated habitats, with cultivated land, settlements, roads, and tourism-related facilities occurring locally. Previous assessments reported local land-use conversion, increased landscape fragmentation, reduced patch connectivity, and human disturbance associated with urban expansion, agriculture, roads, and tourism infrastructure [14]. Tourism-related disturbance has also been recorded in some scenic stream sections of the Zhangjiajie giant salamander system [24]. In the present study, camera traps were deployed mainly in broad-leaved and coniferous forest habitats, and the camera-trap results should therefore be interpreted as reflecting terrestrial mammals and ground-dwelling birds using the monitored forest habitats rather than all habitat types within the reserve.

### 2.2. Camera-Trap Deployment and Data Collection

Camera-trap surveys were conducted from March 2024 to August 2025. To organise sampling and avoid excessive spatial clustering, the reserve was divided into 1 km × 1 km grid cells using ArcGIS 10.8, following commonly used camera-trap monitoring protocols for forest wildlife surveys [25]. The grid system was used as a planning framework, but camera placement followed a grid-assisted targeted design rather than a fully random design; therefore, not every grid cell contained a camera, and selected cells could differ in the number of deployed cameras according to terrain accessibility, habitat conditions, and the presence of wildlife signs. Based on preliminary field surveys, cameras were installed mainly in broad-leaved and coniferous forest habitats at sites likely to record terrestrial mammals and ground-dwelling birds, including animal trails, ridgelines, forest paths, open understory passages, and locations with field signs such as footprints, feces, hair, or feeding traces. This placement strategy was used to improve detection in rugged forest habitats, but it may overrepresent frequently used movement routes and species that preferentially use trails or open passageways [26,27].

A total of 43 infrared camera traps (E3H intelligent detection camera, WiFi standard version, model E3H-V107; sourced from Beijing Prestar Tech. Co., Ltd. [EREAGLE], Beijing, China) were deployed in the reserve. Camera-trap stations were classified according to the dominant land-cover type at each location using the 2020 global 30 m fine land-cover product [28]. Among the 43 stations, 35 were located in broad-leaved forest and 8 in coniferous forest (Figure 1c). The minimum straight-line distance between adjacent cameras was maintained at approximately 300 m, where field conditions allowed, following spacing practices used in previous camera-trap monitoring in mountainous forest habitats [29]. This distance was used to reduce local clustering of sampling points and improve spatial coverage under the constraints of terrain accessibility, forest structure, and equipment safety; it was not intended to ensure individual independence for wide-ranging species.

Cameras were attached to trees or other stable substrates at a height of approximately 0.5–0.8 m above the ground and oriented toward animal trails or open passageways. Direct sunlight, dense foreground vegetation, and locations with a high risk of equipment disturbance were avoided where possible. No bait or lure was used. Each trigger produced three consecutive photographs followed by a 30 s video, with a 10 s interval between successive triggers. According to the manufacturer’s specifications, the cameras had a minimum PIR trigger response time of 0.5 s, a PIR detection distance of up to 20 m, a PIR detection angle of 50°, and a lens field of view of 80° horizontally and 42° vertically. Night-time images were recorded using 940 nm invisible infrared illumination, with an effective illumination distance of up to 30 m. Cameras were checked approximately every three months to replace batteries, exchange memory cards, and verify camera position and function. Because cameras were deliberately placed near wildlife signs and movement routes, detections were interpreted as occurrence records, relative detection frequencies, and activity-time records, rather than as unbiased estimates of absolute abundance or population density. This potential placement bias was considered when interpreting RAI values, naive site occupancy, and diel activity patterns [26,27].

### 2.3. Image Processing and Species Identification

After the memory cards were retrieved, all photographs and videos were organised using BioPhoto V2.1 and sorted by camera station, image or video ID, date, time, and preliminary species identification. Non-target records, including empty triggers, humans, livestock, and domestic animals, were removed before wildlife analyses. Species were identified based on visible external morphological characteristics, such as body size, pelage or plumage pattern, body shape, bill shape, tail shape, posture, and other diagnostic features. Mammal identification mainly followed the Illustrated Handbook of Mammals in China [30], and mammalian taxonomy and nomenclature followed the Catalogue of Mammals in China (2025 Edition) [31]. Bird identification mainly followed Birds of China [32]. Species protection status was determined according to the List of National Key Protected Wild Animals [33] and the IUCN Red List [34]; bird conservation status was also checked against the China Biodiversity Red List: Vertebrates, Volume II: Birds [35].

One camera-trap day was defined as 24 h of continuous operation by a single camera. To reduce pseudo-replication caused by repeated triggers of the same individual or group, consecutive photographs or videos of the same species recorded by the same camera within 30 min were treated as one independent detection event; when different individuals could be clearly distinguished within this interval, they were counted as separate independent records [36]. This 30 min threshold was used as an operational criterion rather than as evidence of complete biological independence. Because movement behaviour and site-use patterns vary among species, this threshold may not fully remove temporal dependence among detections and may influence activity-pattern estimates [37]. Therefore, independent detection events were used to calculate relative detection frequency and summarise activity records, but the results were interpreted cautiously rather than as exact measures of abundance or individual-level activity.

To reduce identification errors, initial species identifications were checked by another trained observer. Records with uncertain or inconsistent identifications were re-examined jointly and assigned to species level only when consensus was reached. Blurred, overexposed, underexposed, partially obstructed, or incomplete images were retained only when species identity could be confidently determined; otherwise, they were classified as unidentified mammal, unidentified bird, or unidentified animal and excluded from species-level analyses. Small rodents that could not be reliably identified to species level from images, except identifiable members of Sciuridae, were also excluded from subsequent statistical analyses. Formal inter-observer agreement statistics were not calculated.

### 2.4. Data Analysis

#### 2.4.1. Completeness of Taxonomic Diversity Identification and Relative Detection Frequency

To evaluate whether the camera-trap survey provided an adequate basis for identifying the taxonomic diversity of terrestrial mammals and ground-dwelling birds in the reserve, sample-based taxonomic completeness curves were generated separately for mammals, ground-dwelling birds, and all identified species combined. Camera-trap stations were used as sampling units. The order of stations was randomised using the specaccum function in the vegan package in R 4.5.2, with 999 random permutations. Mean curves and 95% confidence intervals were used to assess whether the number of detected species tended to approach an asymptote as additional camera-trap stations were included. These curves were used to evaluate the completeness of species detection under the current survey design, rather than to estimate the true total species richness of the reserve.

For each species, we tallied the total number of independent detection events and calculated naive site occupancy (SO), defined as the percentage of camera-trap stations at which the species was recorded at least once [38]. SO was calculated as follows:

(1) $$SO=\frac{S_{i}}{T}\times 100\%\;$$

where SO is the naive site occupancy of species *i*, $S_{i}$ is the number of camera-trap stations where species *i* was detected at least once, and T is the total number of camera-trap stations deployed in the survey.

The relative detection frequency of each species was expressed using the Relative Abundance Index (RAI), calculated as:

(2) $$RAI=\frac{N_{i}}{D}\times 100$$

where RAI is the relative abundance index of species *i*, $N_{i}$ is the total number of independent detection events of species *i* across all camera stations, and D is the total number of effective camera-trap days across all stations. Thus, RAI represents the number of independent detections per 100 camera-trap days.

#### 2.4.2. Diel Activity Patterns, Seasonal Overlap, and Interspecific Temporal Overlap

Diel activity analyses were based on the timestamps of independent detection events. Clock time was converted to solar time using the recording date and the approximate central coordinates of the study area (29.33° N, 110.50° E), allowing activity records to be compared across months with different sunrise and sunset times [39]. Solar time was then converted to radians on a 0–2π scale, with 0:00 and 24:00 treated as the same point in the daily cycle. Because activity data are circular rather than linear, kernel density estimation was used to describe activity distributions without assuming a normal linear distribution of detection times [40].

Focal species for annual activity analysis were selected using a quantitative detection threshold; those with at least 90 independent detections during the full survey period were included in the annual diel activity analyses. Annual activity curves were estimated using circular kernel density methods with the overlap package in R 4.5.2. Bandwidths were selected by getBandwidth (kmax = 3, adjustment = 1) and applied consistently across species. Activity peaks, identified as local maxima of the fitted curves, served for descriptive interpretation only. The annual activity analysis covered 11 species: Chinese ferret-badger (*Melogale moschata*), red-hipped squirrel (*Dremomys pyrrhomerus*), blue whistling thrush (*Myophonus caeruleus*), masked palm civet (*Paguma larvata*), Reeves’s muntjac (*Muntiacus reevesi*), hog badger (*Arctonyx collaris*), golden pheasant (*Chrysolophus pictus*), leopard cat (*Prionailurus bengalensis*), tufted deer (*Elaphodus cephalophus*), rhesus macaque (*Macaca mulatta*), and small Indian civet (*Viverricula indica*).

To quantify the degree of nocturnal activity, a nocturnality index (NI) was calculated for each focal species as the proportion of independent detections recorded during the nighttime period from 18:00 to 06:00 in solar time. For descriptive classification, species were categorised as primarily nocturnal when NI ≥ 0.70, primarily diurnal when NI ≤ 0.30, and mixed or crepuscular when 0.30 < NI < 0.70. These thresholds were used as operational categories to summarise activity tendencies rather than as strict behavioural classifications. Differences in the distribution of day- and night-time detections among broad ecological groups were assessed using Pearson’s chi-squared test.

Seasonal comparisons were based on bioclimatic periods rather than arbitrary calendar groupings. Monthly mean temperature and precipitation data were obtained from NASA POWER for the central coordinates of the study area and are summarised in Table 1 [41]. These data showed that higher temperatures and rainfall were concentrated from May to September, whereas October to April was characterised by lower temperatures and relatively reduced rainfall. Accordingly, May–September was defined as the warm-wet season, corresponding to the main period of higher temperatures, concentrated rainfall, and vegetation growth. October-April was defined as the cool-dry season, corresponding to lower temperatures and reduced rainfall. This two-season classification was used to evaluate whether focal species differed in their activity-density distributions and peak timing between contrasting natural climatic periods.

Seasonal temporal overlap was quantified using the coefficient of overlap (Δ), which ranges from 0, indicating no overlap, to 1, indicating complete overlap. Seasonal overlap analysis was conducted only for species for which both seasonal samples contained more than 75 independent detections. The Δ_4_ estimator was used because the seasonal comparisons were restricted to relatively large samples for which this estimator is recommended. The 95% confidence intervals for Δ_4_ were obtained from 1000 smoothed bootstrap iterations and calculated using bootCI; the basic0 interval was used for reporting [42]. Estimation of Δ_4_, bootstrap resampling, and confidence intervals were performed using the overlapEst, bootstrap, and bootCI functions in the overlap package.

A bootstrap-based peak-shift analysis was used to evaluate whether seasonal changes in activity-density distributions indicated reliable shifts in main activity peak timing for each species. For each bioclimatic period, the main activity peak was identified as the local maximum of the fitted kernel density curve. The difference in peak times between the warm-wet and cool-dry periods was calculated on a circular 24 h scale. Values were constrained between −12 and +12 h. Peak displacement was assessed using 1000 bootstrap resamples. A shift was considered reliable only when the 95% bootstrap confidence interval for displacement did not include zero. This analysis distinguished reliable shifts in peak timing from seasonal differences in the shape, breadth, or concentration of camera-based detections.

Seven species met the seasonal sample-size criterion: blue whistling thrush, red-hipped squirrel, hog badger, masked palm civet, Reeves’s muntjac, golden pheasant, and Chinese ferret-badger.

**Figure 3:**
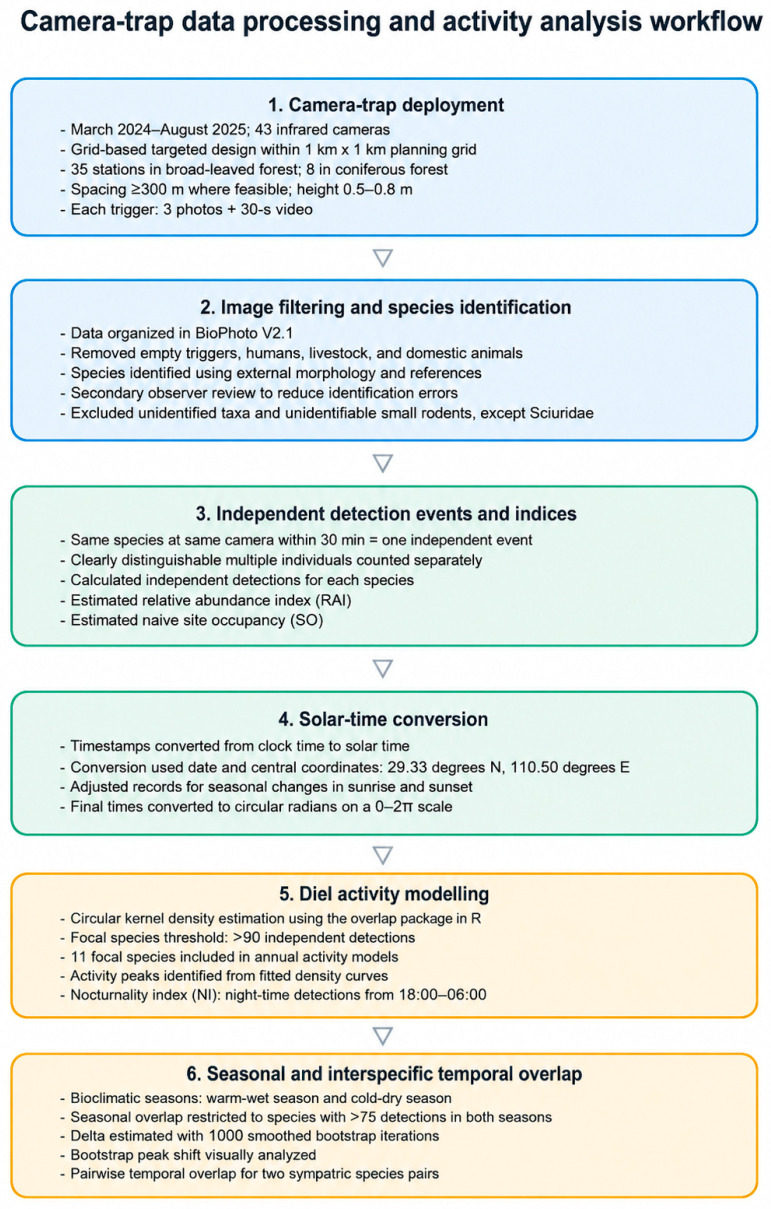
Methodological workflow for camera-trap data processing and ecological analysis. The workflow summarises the six main steps used in this study: (1) grid-assisted targeted deployment of camera traps in broad-leaved and coniferous forest habitats; (2) image and video filtering, species identification, and secondary observer cross-checking using BioPhoto V2.1; (3) extraction of independent detection events using a standardised 30 min operational threshold, followed by calculation of relative abundance index (RAI) and naive site occupancy (SO); (4) conversion of local clock time to solar time and circular radians based on the recording date and central coordinates of the study area; (5) diel activity estimation using non-parametric circular kernel density estimation for focal species with sufficient detections; and (6) seasonal and interspecific temporal overlap analysis using the Δ_4_ estimator, bootstrap confidence intervals, and peak-shift reliability assessment. Arrows indicate the sequential workflow from camera-trap deployment to temporal overlap analysis. Different box colors represent different stages of the analysis: blue boxes indicate data collection and image processing, green boxes indicate detection-event processing and time conversion, and yellow boxes indicate activity modelling and temporal overlap analysis.

Pairwise interspecific temporal overlap analyses were conducted for two ecologically relevant sympatric species pairs: Chinese ferret-badger versus masked palm civet, and Reeves’s muntjac versus tufted deer. These pairs were selected because they were sufficiently detected and represent sympatric terrestrial mammals with potential temporal overlap in forest habitats. The same Δ_4_ estimator and bootstrap procedure described above were used for these pairwise comparisons. These analyses were intended to provide descriptive insights into temporal co-occurrence patterns rather than to test specific ecological mechanisms such as competitive exclusion or niche differentiation. All activity analyses and visualisations were conducted in R 4.5.2 using dplyr 1.2.1, lubridate 1.9.5, ggplot2 4.0.3, patchwork 1.3.2, tidyr 1.3.2, and overlap 0.3.9 (Figure 3).

## 3. Results

### 3.1. Species Composition

Between March 2024 and August 2025, a total of 43 camera-trap stations were established across the survey area, resulting in 16,314 effective camera-trap days. After screening and filtering the image data—removing false triggers and merging consecutive repeated records of the same species at the same monitoring site—3309 independent images suitable for taxonomic identification were retained. Of these, 2180 images (65.88%) were of mammals, and 1129 images (34.12%) were of birds.

From the above dataset, a total of 59 wildlife species were identified, consisting of 18 mammal species and 41 bird species. These are outlined in Table 2 and Table 3. These ambiguous data were excluded from subsequent analyses.

#### 3.1.1. Mammal Composition

A total of 18 mammal species were recorded, belonging to 5 orders and 9 families. The order Carnivora was the most species-rich, with seven species, accounting for 38.89% of the total mammal species, followed by Rodentia, which included 6 species (33.33%). The order Cetartiodactyla was represented by three species (16.67%), and Primates and Lagomorpha each contributed one species (5.56%).

Regarding conservation status, the small Indian civet was listed as a Class I National Key Protected Wild Animal in China. Three species were classified as Class II National Key Protected Wild Animals: the rhesus macaque, leopard cat, and tufted deer. Additionally, one Chinese endemic species was recorded: the Reeves’s muntjac. According to the China Biodiversity Red List, one species—leopard cat—was classified as Vulnerable (VU), while seven species were classified as Near Threatened (NT): the ferret-badger, hog badger, small Indian civet, masked palm civet, Reeves’s muntjac, tufted deer, and red-hipped squirrel. The IUCN Red List classified the hog badger as Vulnerable (VU), and both the yellow-bellied weasel (*Mustela kathiah*) and tufted deer were listed as Near Threatened (NT).

The five mammal species with the highest relative abundance index (RAI) values were, in descending order: ferret-badger (2.49), red-hipped squirrel (2.35), masked palm civet (1.96), Reeves’s muntjac (1.72), and hog badger (1.70).

**Figure 4:**
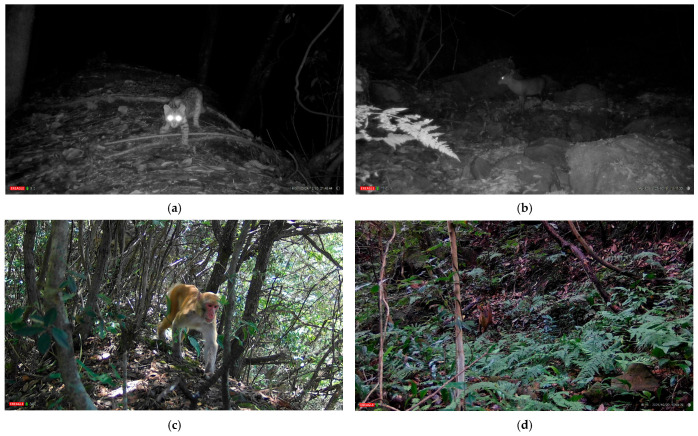
Infrared camera trap images of (**a**) leopard cat, (**b**) tufted deer, (**c**) rhesus macaque, and (**d**) Reeves’s muntjac at the Zhangjiajie Chinese Giant Salamander National Nature Reserve from March 2024 to August 2025.

Representative camera-trap images of selected mammal species recorded during the survey are shown in Figure 4.

#### 3.1.2. Bird Composition

A total of 41 bird species were recorded, representing 6 orders and 18 families. The order Passeriformes dominated, with 31 species, accounting for 75.61% of all bird species. Galliformes included six species (14.63%), and Columbiformes, Strigiformes, Accipitriformes, and Piciformes each contained one species (2.44%).

Regarding conservation status, one bird species, Elliot’s pheasant (*Syrmaticus ellioti*), was listed as a Class I National Key Protected Wild Animal in China. Ten species were classified as Class II National Key Protected Wild Animals, including the silver pheasant (*Lophura nycthemera*), Temminck’s tragopan (*Tragopan temminckii*), golden pheasant, koklass pheasant (*Pucrasia macrolopha*), Himalayan owl (*Strix nivicolum*), crested serpent eagle (*Spilornis cheela*), fairy pitta (*Pitta nympha*), chestnut-crowned laughingthrush (*Pterorhinus berthemyi*), red-billed leiothrix (*Leiothrix lutea*), and Chinese hwamei (*Garrulax canorus*). Five bird species were Chinese endemics: Elliot’s pheasant, golden pheasant, Chinese bamboo partridge (*Bambusicola thoracicus*), chestnut-crowned laughingthrush, and Kessler’s thrush (*Turdus mupinensis*).

According to the China Biodiversity Red List, two bird species—Elliot’s pheasant and fairy pitta—were classified as Vulnerable (VU), while six species were classified as Near Threatened (NT): Temminck’s tragopan, golden pheasant, Himalayan owl, crested serpent eagle, Chinese hwamei, and Kessler’s thrush. The IUCN Red List classified the fairy pitta as Vulnerable (VU), with all other species listed as Least Concern (LC).

The 5 bird species with the highest RAI values were, in descending order: blue whistling thrush (2.31), golden pheasant (1.34), Red-flanked Bluetail (*Tarsiger cyanurus*, 0.50), Collared Laughingthrush (*Pterorhinus pectoralis*, 0.42), and Elliot’s pheasant (0.38).

**Figure 5:**
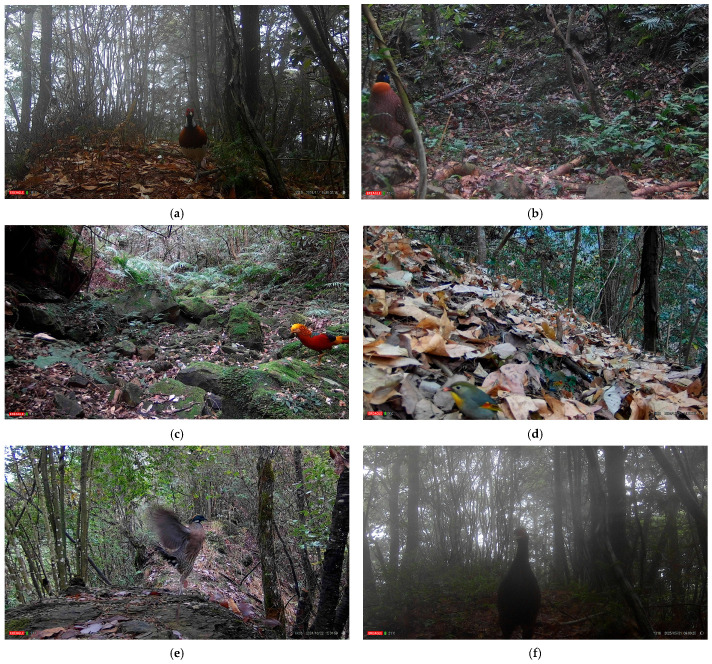
Infrared camera trap images of (**a**) Elliot’s pheasant, (**b**) Temminck’s tragopan, (**c**) golden pheasant, (**d**) Red-billed leiothrix, (**e**) Koklass pheasant, and (**f**) Silver pheasant at the Zhangjiajie Chinese Giant Salamander National Nature Reserve from March 2024 to August 2025.

Representative camera-trap images of selected ground-dwelling bird species recorded during the survey are shown in Figure 5.

### 3.2. Completeness of Taxonomic Diversity Identification

**Figure 6:**
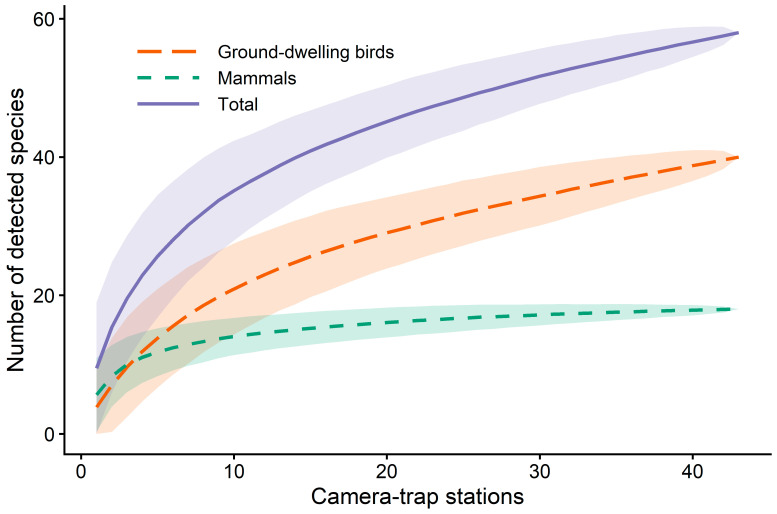
Taxonomic completeness curves for mammals, ground-dwelling birds, and all detected species recorded by camera traps in the Hunan Zhangjiajie Giant Salamander National Nature Reserve. Camera-trap stations were used as sampling units. Lines represent mean detection curves based on 999 random permutations, and shaded areas indicate 95% confidence intervals.

The taxonomic completeness curves, generated using camera-trap stations as sampling units, showed the pattern of species detection under the current survey design (Figure 6). For mammals, ground-dwelling birds, and all identified species combined, the number of recorded species increased rapidly during the early stage of sampling and then gradually tended to approach an asymptote as additional camera-trap stations were included. This pattern indicates that the survey effort provided an adequate basis for documenting the main camera-detectable terrestrial mammals and ground-dwelling birds in the reserve.

The mammal curve approached an asymptote under the current sampling conditions and reached 18 identified species. The ground-dwelling bird curve increased more gradually and reached 41 identified species, suggesting that broader spatial coverage across camera-trap stations was needed to characterise this group. The combined curve reached 59 identified species and also tended to stabilise toward the end of the sampling sequence. These results indicate that the current camera-trap survey captured most of the camera-detectable mammal and ground-dwelling bird taxa under the present sampling design, but they should not be interpreted as evidence that the total vertebrate diversity of the reserve was completely inventoried.

### 3.3. Activity Pattern Models and Seasonal Overlap

#### 3.3.1. Annual Diel Activity Patterns of Focal Species

**Figure 7:**
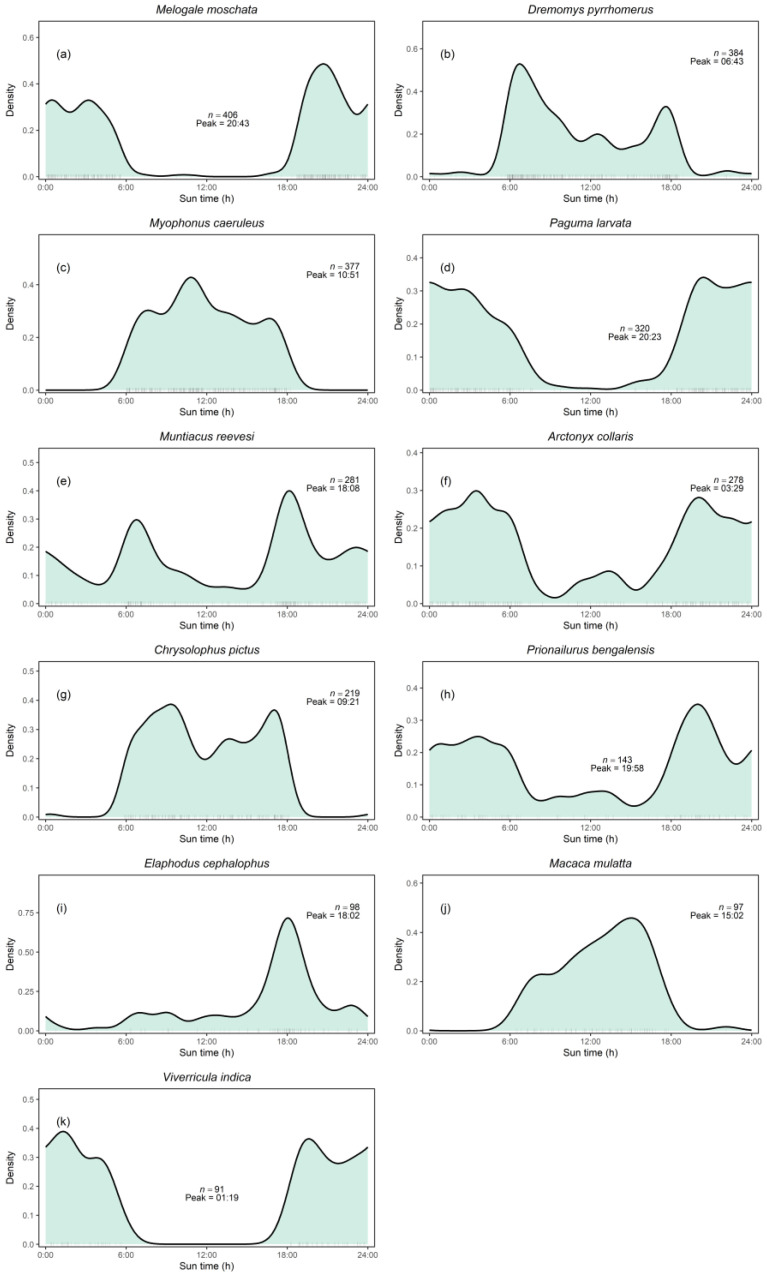
Annual diel activity patterns of 11 focal species recorded by camera traps in the Hunan Zhangjiajie Giant Salamander National Nature Reserve. Activity curves were estimated using kernel density estimation based on solar time. Shaded areas indicate fitted activity density, and rug marks indicate independent detection events. The number of independent detections and the main activity peak are shown for each species. Subfigures represent the diel activity patterns of the following species: (**a**) *Melogale moschata*; (**b**) *Dremomys pyrrhomerus*; (**c**) *Myophonus caeruleus*; (**d**) *Paguma larvata*; (**e**) *Muntiacus reevesi*; (**f**) *Arctonyx collaris*; (**g**) *Chrysolophus pictus*; (**h**) *Prionailurus bengalensis*; (**i**) *Elaphodus cephalophus*; (**j**) *Macaca mulatta*; and (**k**) *Viverricula indica*. Solid lines indicate the fitted kernel density curves of diel activity patterns for each species.

Annual diel activity patterns were estimated for 11 focal species with at least 90 independent detections during the survey period (Figure 7). The fitted kernel density curves showed clear differences in activity allocation across the 24 h solar-time cycle, with main activity peaks ranging from early morning to late evening.

The carnivores showed the highest nocturnality indices (NI), calculated as the proportion of independent detections recorded during the nighttime period from 18:00 to 06:00 in solar time. Small Indian civet (*n* = 91, peak = 01:19, NI = 1.000), Chinese ferret-badger (*n* = 406, peak = 20:43, NI = 0.983), masked palm civet (*n* = 320, peak = 20:23, NI = 0.897), hog badger (*n* = 278, peak = 03:29, NI = 0.791), and leopard cat (*n* = 143, peak = 19:58, NI = 0.790) were predominantly nocturnal.

By contrast, the red-hipped squirrel (*n* = 384, peak = 06:43, NI = 0.125), golden pheasant (*n* = 219, peak = 09:21, NI = 0.041), rhesus macaque (*n* = 97, peak = 15:02, NI = 0.010), and blue whistling thrush (*n* = 377, peak = 10:51, NI = 0.005) were primarily diurnal. Reeves’s muntjac (*n* = 281, peak = 18:08, NI = 0.505) and tufted deer (*n* = 98, peak = 18:02, NI = 0.469) showed mixed or crepuscular-to-nocturnal tendencies, with activity concentrated around dusk.

At the ecological-group level, the proportion of day- and night-time detections differed significantly among carnivores, ungulates, the squirrel, ground-dwelling birds, and primate groups (Pearson’s χ^2^ test: χ^2^ = 1640.7, df = 4, *p* < 0.001). Carnivores were strongly biased toward night-time activity, whereas ground-dwelling birds, the squirrel, and the macaque were mainly diurnal. These results indicate pronounced temporal differentiation among the dominant camera-detected taxa in the reserve. However, the activity curves and NI values represent camera-based detection patterns under the current sampling design and should not be interpreted as complete representations of all behavioural activity.

#### 3.3.2. Seasonal Temporal Overlap and Peak-Shift Reliability of Focal Species

**Figure 8:**
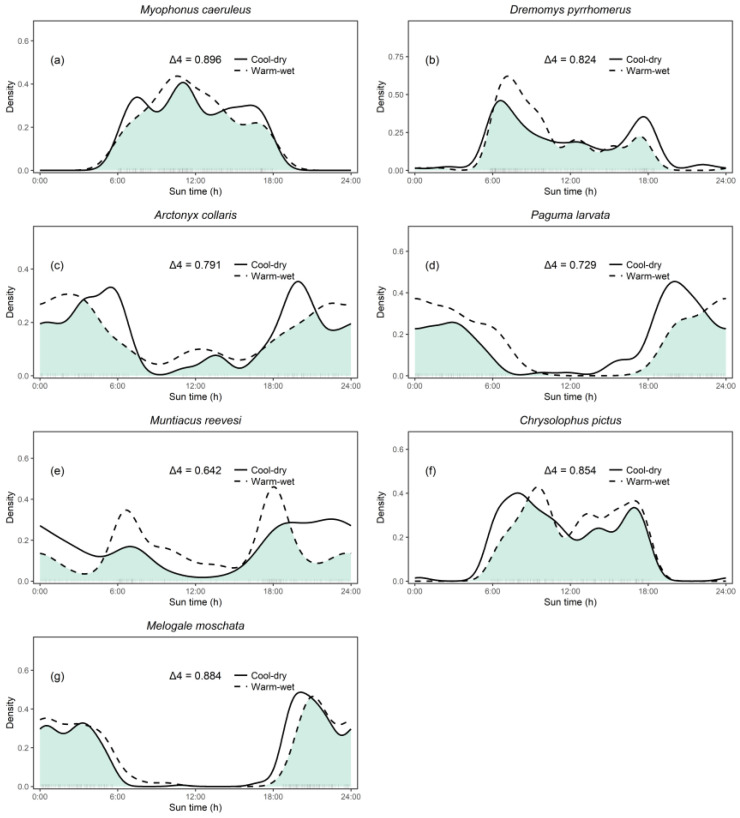
Seasonal temporal overlap of diel activity-density distributions for seven focal species between the cool-dry and warm-wet seasons. Solid lines represent activity density during the cool-dry season, dashed lines represent activity density during the warm-wet season, and shaded areas indicate the overlap between the two seasonal activity distributions. Δ_4_ values indicate the coefficient of overlap estimated from independent detection events based on solar time. Subfigures represent seasonal diel activity overlap for the following species: (**a**) *Myophonus caeruleus*; (**b**) *Dremomys pyrrhomerus*; (**c**) *Arctonyx collaris*; (**d**) *Paguma larvata*; (**e**) *Muntiacus reevesi*; (**f**) *Chrysolophus pictus*; and (**g**) *Melogale moschata*. Rug marks along the x-axis indicate the times of independent detection records used to estimate the activity curves.

Among the 11 focal species included in the annual diel activity analysis, 7 had sufficient independent detections in both the cool-dry and warm-wet seasons and were therefore included in the seasonal temporal overlap analysis: blue whistling thrush, red-hipped squirrel, hog badger, masked palm civet, Reeves’s muntjac, golden pheasant, and Chinese ferret-badger (Figure 8, Table 4). The seasonal overlap coefficients ranged from 0.642 to 0.896, indicating interspecific variation in the similarity of camera-based activity-density distributions between the two bioclimatic periods.

Blue whistling thrush showed the highest seasonal temporal overlap (Δ_4_ = 0.896, 95% CI: 0.821–0.954), followed by Chinese ferret-badger (Δ_4_ = 0.884, 95% CI: 0.805–0.949), golden pheasant (Δ_4_ = 0.854, 95% CI: 0.765–0.935), and red-hipped squirrel (Δ_4_ = 0.824, 95% CI: 0.745–0.896). Hog badger showed an intermediate level of seasonal overlap (Δ_4_ = 0.791, 95% CI: 0.704–0.873), whereas masked palm civet (Δ_4_ = 0.729, 95% CI: 0.639–0.820) and Reeves’s muntjac (Δ_4_ = 0.642, 95% CI: 0.548–0.736) showed lower overlap values. These lower values indicate seasonal differences in the shape or concentration of activity-density distributions rather than, by themselves, reliable shifts in the timing of activity peaks.

To evaluate whether seasonal differences in the curves reflected reliable displacement of daily activity peaks, bootstrap-based peak-shift analyses were conducted. For six of the seven species, the 95% confidence intervals of peak displacement included zero, indicating no statistically reliable seasonal shift in their main activity peaks. For example, the main peak of blue whistling thrush shifted slightly from 11:02 in the cool-dry season to 10:34 in the warm-wet season, and that of red-hipped squirrel shifted from 06:37 to 07:11; however, these shifts were not reliable based on bootstrap confidence intervals. Similar non-reliable peak displacement was found for hog badger, Reeves’s muntjac, golden pheasant, and Chinese ferret-badger.

Masked palm civet was the only species showing reliable seasonal displacement of its main activity peak, shifting from 20:03 in the cool-dry season to 00:03 in the warm-wet season, with a bootstrap 95% CI for peak displacement of 0.47–7.19 h. Overall, most focal species retained broadly similar peak activity times between the two bioclimatic periods. Therefore, seasonal differences in overlap coefficients should be interpreted primarily as differences in the shape, breadth, or concentration of camera-based detections within the diel cycle, rather than as direct evidence of seasonal changes in biological activity rhythms. These results should not be interpreted as evidence of behavioural adaptation, population-density change, or causal responses to environmental drivers without additional ecological or demographic data.

#### 3.3.3. Pairwise Interspecific Temporal Overlap

**Figure 9:**
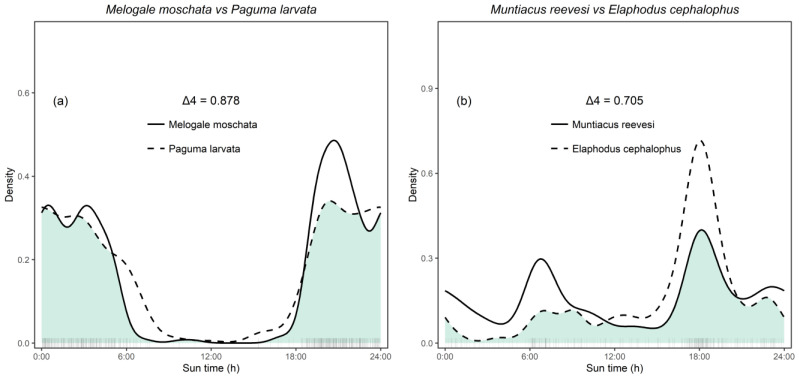
Pairwise interspecific temporal overlap between sympatric species pairs. Solid and dashed lines represent fitted activity-density curves for each species, and shaded areas indicate the temporal overlap between paired species. Δ_4_ values indicate the coefficient of overlap estimated from independent detection events based on solar time. Panel (**a**) shows Chinese ferret-badger and masked palm civet; panel (**b**) shows Reeves’s muntjac and tufted deer.

Pairwise interspecific temporal overlap was assessed for two sympatric species pairs: Chinese ferret-badger versus masked palm civet, and Reeves’s muntjac versus tufted deer (Figure 9). The overlap coefficient was higher for Chinese ferret-badger and masked palm civet (Δ_4_ = 0.878, 95% CI: 0.832–0.923) than for Reeves’s muntjac and tufted deer (Δ_4_ = 0.705, 95% CI: 0.607–0.801).

Both Chinese ferret-badger and masked palm civet were mainly nocturnal, and their activity-density curves showed broad overlap during the nighttime period. In contrast, Reeves’s muntjac and tufted deer both showed activity concentrated around dusk, but the tufted deer exhibited a sharper evening peak, whereas Reeves’s muntjac showed a broader activity distribution extending into other parts of the diel cycle. These results indicate that the two species pairs differed in the degree and shape of their temporal overlap.

However, temporal overlap should be interpreted cautiously. Similar activity timing does not necessarily indicate direct interaction, competition, or shared space use. Spatial segregation among species may still occur even when their diel activity patterns overlap. In addition, because camera traps were placed along animal trails and other signs of wildlife activity, shared use of frequently travelled routes may inflate apparent temporal overlap estimates. Therefore, the pairwise overlap results are best interpreted as descriptive evidence of temporal co-occurrence patterns under the current camera-trap design, rather than as direct evidence of ecological interaction or niche similarity.

## 4. Discussion

This study shows that Hunan Zhangjiajie Giant Salamander National Nature Reserve supports a camera-detected terrestrial vertebrate assemblage composed of mammals and ground-dwelling birds with different ecological roles and habitat associations. Although the reserve was established primarily for the conservation of the Chinese giant salamander and associated aquatic ecosystems, the present results indicate that the monitored broad-leaved and coniferous forest habitats also provide important habitat for terrestrial wildlife. The detected assemblage included small and medium-sized carnivores, ungulates, squirrels, primates, pheasants, thrushes, laughingthrushes, and other ground-dwelling or understory-associated birds. These findings suggest that the biodiversity value of the reserve extends beyond its original aquatic conservation focus. However, because the camera traps in this study were deployed only in broad-leaved and coniferous forests, the results should be interpreted as evidence for the conservation value of the monitored forest habitats rather than as a reserve-wide assessment of all habitat types.

The community pattern recorded in this study is broadly comparable with infrared-camera surveys conducted in other subtropical protected areas of Hunan and the Wuling Mountain region. The most geographically relevant comparison is Hunan Gaowangjie National Nature Reserve, where camera-trap monitoring also documented a diverse assemblage of mammals and birds in a subtropical forest landscape, particularly medium-sized mammals and ground-dwelling birds [11]. Similar surveys in Hunan Dawei Mountain, Badagongshan National Nature Reserve, and the Jintongshan Region of Hunan Nanshan National Park also reported forest-associated mammal and bird communities comparable to the camera-detected assemblage recorded in the present study [43,44]. These regional comparisons suggest that the Zhangjiajie reserve shares several typical features of subtropical forest camera-detected communities in Hunan, including the frequent occurrence of mesocarnivores, ungulates, squirrels, pheasants, and understory birds. However, its conservation significance is distinctive because these terrestrial forest communities occur within a protected area whose management has historically focused more strongly on an aquatic flagship species. Therefore, conservation planning in the reserve should integrate the protection of Chinese giant salamander habitats with increased attention to broad-leaved and coniferous forest habitats used by terrestrial mammals and ground-dwelling birds.

The detection of Chinese endemic and nationally protected species further highlights the regional conservation importance of the reserve’s forest habitats. Among mammals, Reeves’s muntjac was the only Chinese endemic species recorded, but several protected or conservation-concern species were also detected, including small Indian civet, rhesus macaque, leopard cat, and tufted deer. Among birds, the records included several Chinese endemic pheasants and passerines, such as Elliot’s pheasant, golden pheasant, Chinese bamboo partridge, chestnut-crowned laughingthrush, and Kessler’s thrush. These species indicate that the monitored forest habitats contribute not only to local biodiversity maintenance but also to the conservation of endemic and protected taxa within the broader subtropical mountain landscape of Hunan.

The station-level occurrence patterns summarised by RAI and naive site occupancy provide insight into how wildlife used the monitored portion of the reserve (Table 2 and Table 3). Species with broader station occurrence and higher detection frequency likely represent common or regularly detected components of the broad-leaved and coniferous forest camera-trap network, whereas species recorded at only a few stations may reflect localised habitat use, low local abundance, specialised microhabitat requirements, or lower detectability under the current survey design. Accordingly, RAI and naive site occupancy should be interpreted as relative indicators of detection frequency and station-level occurrence, not as estimates of population density or complete spatial distributions across the entire reserve. Although formal detection-corrected occupancy models were not fitted in this study, the naive site occupancy values provide a useful first description of station-level occurrence. Future studies using repeated-occasion detection histories, site-level covariates, and broader spatial coverage could apply occupancy or community-occupancy models to estimate detection-corrected occurrence and species richness more robustly [38,45,46].

The mammal community recorded by camera traps was dominated by mesopredators, small and medium-sized carnivores, ungulates, squirrels, and primates. Chinese ferret-badger, masked palm civet, hog badger, leopard cat, and small Indian civet represented the main carnivore component detected in the monitored forest habitats. This composition suggests that forest trails, understory passages, and other ground-level movement routes are regularly used by small and medium-sized carnivores with different feeding habits, including omnivorous mustelids, viverrids, and small felids. These species may play important ecological roles in seed dispersal, predation on small vertebrates and invertebrates, carrion use, and the regulation of small-animal communities. Their relatively frequent detection also suggests that small and medium-sized carnivores may be useful focal taxa for future long-term monitoring of forest habitat quality and disturbance pressure in the reserve.

The absence of large apex predators from the present camera-trap records is noteworthy in a broader conservation context. Protected areas that still retain large carnivores, such as tiger (*Panthera tigris*), leopard (*Panthera pardus*), Asian golden cat (*Catopuma temminckii*), dhole (*Cuon alpinus*), wolf (*Canis lupus*), or snow leopard (*Panthera uncia*), may represent more complete trophic systems and may differ from mesopredator-dominated communities in species composition, relative detection frequencies, and activity patterns [47,48]. However, direct comparison of RAI values among protected areas should be made cautiously, because RAI is strongly affected by camera placement, survey effort, habitat coverage, detection probability, and species-specific movement behaviour. Therefore, the present study cannot determine whether the mammal and ground-dwelling bird community in Zhangjiajie is statistically different from communities in protected areas that still retain large carnivores. Nevertheless, the lack of records of large apex predators, together with the relatively frequent detection of small and medium-sized carnivores, suggests that the carnivore assemblage detected in the monitored forest habitats was dominated by mesopredators. This pattern may reflect regional differences in trophic structure, historical disturbance, habitat fragmentation, low densities of large carnivores, or the limited spatial coverage of the present survey, but it should not be interpreted as evidence of complete absence without broader landscape-scale monitoring. Future studies using standardised camera-trap designs across multiple protected areas would be needed to test whether reserves with and without large carnivores differ consistently in mammal and ground-dwelling bird community composition, RAI structure, and diel activity patterns.

The bird records should be interpreted in light of the inherent sampling limitations of ground-based camera traps. The species recorded in this study mainly represent birds that use the forest floor, understory, trails, and other ground-level spaces within the camera views. In contrast, canopy-dwelling species, shrub-layer birds moving through dense vegetation, and highly mobile vocal species that rarely descend to the ground were likely underrepresented. Because cameras were placed near animal signs and movement routes, trail-using species may also have been detected more frequently than species moving more diffusely through closed understory vegetation [5,26]. In addition, detectability varies among species according to body size, movement speed, flocking behaviour, activity level, and the height or angle at which individuals pass in front of the camera. Therefore, the bird assemblage reported here should be regarded as a camera-detected subset of ground-dwelling and understory-associated birds, rather than a complete inventory of the reserve’s avifauna. Future surveys should integrate camera trapping with line transects, point counts, passive acoustic monitoring, and targeted surveys of canopy and shrub-layer birds to obtain a more complete assessment of the bird community [49].

The bird assemblage was characterised by a strong representation of ground-dwelling and understory-associated species, especially pheasants, thrushes, laughingthrushes, and other forest-floor or low-stratum birds. The detection of multiple pheasant species, including Elliot’s pheasant, golden pheasant, silver pheasant, Temminck’s tragopan, koklass pheasant, and Chinese bamboo partridge, indicates that the monitored forest habitats provide important ground-level resources and cover for Galliformes. Pheasants are often sensitive to hunting pressure, understory structure, and forest disturbance, and their presence in the reserve suggests that the forest floor retains habitat value for conservation-relevant ground birds. Together with the records of forest-floor thrushes and laughingthrushes, these findings indicate that the monitored broad-leaved and coniferous forests support a functionally diverse ground-dwelling bird assemblage.

The diel activity results further indicate temporal structuring among the main ecological groups recorded in the reserve. Carnivores were predominantly nocturnal, whereas squirrels, ground-dwelling birds, and macaques were mainly diurnal, and ungulates showed more mixed or crepuscular-to-nocturnal tendencies. This pattern, supported by the nocturnality index and the comparison of day- and night-time detections among ecological groups, suggests that different taxa use the daily cycle in different ways. Such temporal differentiation may reflect differences in foraging strategy, sensory ecology, predator avoidance, thermoregulation, and sensitivity to human disturbance [20,50,51]. Nocturnal activity in small and medium-sized carnivores could be associated with prey-searching behaviour, avoidance of daytime disturbance, or cooler thermal conditions, whereas the daytime activity of ground-dwelling birds and squirrels may be linked to visual foraging and movement in forest-floor and understory environments [50,52]. Ungulates may use crepuscular or nocturnal periods to balance forage acquisition, thermal conditions, and exposure risk. However, these interpretations should remain cautious because camera-based activity curves describe detection patterns under the current survey design and cannot by themselves identify the causal mechanisms driving animal behaviour.

Seasonal comparisons should be interpreted as differences in camera-based activity-density distributions rather than as direct evidence of broad seasonal changes in biological activity rhythms. Although seasonal activity analyses can reveal biologically meaningful variation in time use, camera-trap data alone cannot identify the environmental mechanisms driving these patterns without additional information on food resources, temperature, reproduction, habitat use, and human disturbance [53,54]. In the present study, most focal species retained broadly similar main activity peaks between the cool-dry and warm-wet seasons, indicating that seasonal differences in the curves may mainly reflect changes in the breadth, intensity, or concentration of detections within the same general daily activity window. The masked palm civet was the only focal species with a reliable seasonal peak displacement, suggesting that this species may have greater seasonal flexibility in activity timing. This pattern could be associated with seasonal changes in food availability, habitat use, or route selection, as previous research has shown that masked palm civets can shift diet and foraging sites in relation to fruit availability [55]. Nevertheless, these mechanisms cannot be confirmed without direct dietary, environmental, reproductive, or spatial-use data. Therefore, the seasonal activity results should be treated as evidence of variation in detection-based temporal distributions, not as proof of behavioural adaptation, migration, or population-density change.

The pairwise temporal overlap results should also be interpreted cautiously in relation to potential interspecific interactions. The overlap between Chinese ferret-badger and masked palm civet indicates that these two nocturnal mesocarnivores used similar portions of the daily cycle, whereas Reeves’s muntjac and tufted deer shared activity mainly around crepuscular or evening periods. However, similarity in activity timing does not necessarily indicate direct interaction, competition, or niche similarity [42,51]. Species may overlap temporally while remaining spatially separated by habitat type, elevation, slope position, vegetation structure, or microhabitat use. Conversely, because cameras were often placed along animal trails and other movement routes, repeated use of the same paths by multiple species may increase apparent temporal overlap [26]. Thus, the observed pairwise overlap patterns are best interpreted as descriptive evidence of temporal co-occurrence within the camera-trap network, rather than as proof of direct ecological interaction or coexistence mechanisms.

These results also provide practical implications for reserve management. Because many carnivores and some ungulates were mainly detected at night or around dusk, intensive human activities in monitored forest habitats should be minimised during these periods. Night-time disturbance from patrol lighting, vehicle movement, construction, and tourism-related activities should be restricted, especially in broad-leaved and coniferous forest areas where wildlife detections were concentrated. Patrol schedules could be adjusted according to daily and seasonal activity patterns: daytime patrols may be more suitable for monitoring ground-dwelling birds and diurnal mammals, whereas nocturnal mammals should be monitored primarily through passive camera traps rather than frequent nighttime human access. In addition, both broad-leaved and coniferous forests should be maintained as important terrestrial habitats for mammals and ground-dwelling birds within the reserve. Tourism management should limit off-trail movement, artificial lighting, and nighttime access in forest areas with frequent wildlife detections. Long-term monitoring should retain fixed camera stations for temporal comparison while adding stratified or randomised camera locations across different forest types, elevations, and distances from trails to reduce sampling bias and improve inference about spatial occurrence.

## 5. Conclusions

While this study provides valuable baseline information on the terrestrial mammals and ground-dwelling birds detected in the monitored broad-leaved and coniferous forest habitats of Hunan Zhangjiajie Giant Salamander National Nature Reserve, several limitations remain. First, the lack of direct comparisons with standardised camera-trap datasets from other protected areas in the Wuling Mountain region limits our ability to evaluate how distinctive the reserve’s terrestrial vertebrate assemblage is at a broader biogeographical scale. Second, because camera traps were deployed mainly in broad-leaved and coniferous forests and were placed near animal trails, forest paths, open understory passages, and wildlife signs, the survey may have underestimated species that use other habitat types, canopy strata, dense shrub layers, or areas away from trails. Third, RAI and naive site occupancy provide useful descriptions of relative detection frequency and station-level occurrence, but they do not account for imperfect detection and should not be interpreted as estimates of abundance, density, or true occupancy. Despite these limitations, this study establishes an important baseline for long-term monitoring and multi-taxon conservation management in a protected area historically focused on the Chinese giant salamander and aquatic ecosystem conservation. Based on our findings, we make the following recommendations: (1) broad-leaved and coniferous forest habitats should be maintained as important terrestrial wildlife habitats within the reserve, especially in areas where endemic, nationally protected, or threatened species were detected; (2) unnecessary human disturbance, including night-time lighting, vehicle movement, off-trail activities, and tourism-related disturbance, should be reduced during sensitive activity periods, particularly dusk, night, and early morning when many mammals are active; (3) long-term camera-trap monitoring should retain fixed stations for temporal comparison while adding stratified or randomised camera locations across different forest types, elevations, disturbance gradients, and unsampled habitat types; and (4) future studies should incorporate repeated-occasion detection histories, environmental covariates, and detection-corrected occupancy or community-occupancy models to better assess species occurrence, habitat associations, and changes in terrestrial vertebrate communities over time.

## Figures and Tables

**Table 1:** Monthly climatic characteristics used to define bioclimatic periods in the Zhangjiajie region.

Month	Mean Temperature (°C)	Precipitation (mm)	Bioclimatic Period
January	2.6	1.2	cool-dry season
February	5.2	1.9	cool-dry season
March	10.2	2.9	cool-dry season
April	15.5	5	cool-dry season
May	19.7	6.7	warm-wet season
June	23.3	7.5	warm-wet season
July	25.6	7.4	warm-wet season
August	24.9	5.1	warm-wet season
September	21.1	3.9	warm-wet season
October	15.7	3.2	cool-dry season
November	10.2	2.4	cool-dry season
December	4.2	0.8	cool-dry season

Note: Values were derived from NASA POWER monthly climate data for the same central coordinates of the study area. Mean temperature represents the monthly mean 2 m air temperature, and precipitation represents the monthly total precipitation. May–September was defined as the warm-wet season, whereas October–April was defined as the cool-dry season based on the combined seasonal patterns of temperature and precipitation.

**Table 2:** Checklist of 18 mammal species recorded in the Zhangjiajie Chinese Giant Salamander National Nature Reserve, Hunan, China.

No.	Order	Family	Common Names	Scientific Name	National Category	China Red List	IUCN Red List	RAI	SO (%)	Detection Counts
1	Primates	Cercopithecidae	Rhesus macaque	*Macaca mulatta*	II	LC	LC	0.60	32.56	97
2	Carnivora	Mustelidae	Yellow-bellied weasel	*Mustela kathiah*	-	LC	NT	0.13	16.28	21
3	Carnivora	Mustelidae	Siberian weasel	*Mustela sibirica*	-	LC	LC	0.14	25.58	23
4	Carnivora	Mustelidae	Chinese ferret-badger	*Melogale moschata*	-	NT	LC	2.49	69.77	406
5	Carnivora	Mustelidae	Greater hog-badger	*Arctonyx collaris*	-	NT	VU	1.70	62.79	278
6	Carnivora	Viverridae	Small Indian civet	*Viverricula indica*	I	NT	LC	0.56	20.93	91
7	Carnivora	Viverridae	Masked palm civet	*Paguma larvata*	-	NT	LC	1.96	69.77	320
8	Carnivora	Felidae	Leopard cat	*Prionailurus bengalensis*	II	VU	LC	0.88	65.12	143
9	Cetartiodactyla	Suidae	Wild boar	*Sus scrofa*	-	LC	LC	0.03	6.98	5
10	Cetartiodactyla	Cervidae	Reeves’s muntjac	*Muntiacus reevesi* *	-	NT	LC	1.72	34.88	281
11	Cetartiodactyla	Cervidae	Tufted deer	*Elaphodus cephalophus*	II	NT	NT	0.60	62.79	98
12	Rodentia	Sciuridae	Pallas’s squirrel	*Callosciurus erythraeus*	-	LC	LC	0.06	13.95	9
13	Rodentia	Sciuridae	Maritime striped squirrel	*Tamiops maritimus*	-	LC	LC	0.01	2.33	2
14	Rodentia	Sciuridae	Swinhoe’s striped squirrel	*Tamiops swinhoei*	-	LC	LC	0.09	6.98	14
15	Rodentia	Sciuridae	Red-hipped squirrel	*Dremomys pyrrhomerus*	-	NT	LC	2.35	60.47	384
16	Rodentia	Sciuridae	Red and white giant flying squirrel	*Petaurista alborufus*	-	LC	LC	0.02	4.65	4
17	Rodentia	Spalacidae	Chinese bamboo rat	*Rhizomys sinensis*	-	LC	LC	0.01	4.65	2
18	Lagomorpha	Leporidae	Chinese hare	*Lepus sinensis*	-	LC	LC	0.01	2.33	2

Note: * indicates species endemic to China. National protection categories: I = Class I nationally protected wildlife; II = Class II nationally protected wildlife; - = not listed. China Vertebrate Red List and IUCN Red List categories: LC = Least Concern; NT = Near Threatened; VU = Vulnerable. RAI = relative abundance index. SO, naive site occupancy, calculated as the percentage of camera-trap stations at which a species was detected at least once.

**Table 3:** Checklist of 41 bird species recorded in the Zhangjiajie Chinese Giant Salamander National Nature Reserve, Hunan, China.

No.	Order	Family	Common Names	Scientific Name	National Category	China Red List	IUCN Red List	RAI	SO (%)	Detection Counts
1	Galliformes	Phasianidae	Elliot’s pheasant	*Syrmaticus ellioti* *	I	VU	LC	0.38	30.23	62
2	Galliformes	Phasianidae	Silver pheasant	*Lophura nycthemera*	II	LC	LC	0.01	4.65	2
3	Galliformes	Phasianidae	Temminck’s tragopan	*Tragopan temminckii*	II	NT	LC	0.13	6.98	20
4	Galliformes	Phasianidae	Golden pheasant	*Chrysolophus pictus* *	II	NT	LC	1.34	30.23	219
5	Galliformes	Phasianidae	Chinese bamboo partridge	*Bambusicola thoracicus* *	-	LC	LC	0.20	18.60	33
6	Galliformes	Phasianidae	Koklass pheasant	*Pucrasia macrolopha*	II	LC	LC	0.15	11.63	25
7	Columbiformes	Columbidae	Oriental turtle-dove	*Streptopelia orientalis*	-	LC	LC	0.22	20.93	36
8	Strigiformes	Strigidae	Himalayan owl	*Strix nivicolum*	II	NT	LC	0.01	2.33	1
9	Accipitriformes	Accipitridae	Crested serpent-eagle	*Spilornis cheela*	II	NT	LC	0.03	4.65	5
10	Piciformes	Picidae	Grey-headed woodpecker	*Picus canus*	-	LC	LC	0.01	2.33	2
11	Passeriformes	Pittidae	Fairy pitta	*Pitta nympha*	II	VU	VU	0.01	2.33	1
12	Passeriformes	Corvidae	Red-billed blue magpie	*Urocissa erythroryncha*	-	LC	LC	0.12	13.95	19
13	Passeriformes	Corvidae	Eurasian jay	*Garrulus glandarius*	-	LC	LC	0.01	2.33	1
14	Passeriformes	Paridae	Japanese tit	*Parus minor*	-	LC	NE	0.01	2.33	1
15	Passeriformes	Paridae	Green-backed tit	*Parus monticolus*	-	LC	LC	0.01	2.33	1
16	Passeriformes	Cisticolidae	Yellow-bellied prinia	*Prinia flaviventris*	-	LC	LC	0.02	2.33	1
17	Passeriformes	Pycnonotidae	Brown-breasted bulbul	*Pycnonotus xanthorrhous*	-	LC	LC	0.02	2.33	4
18	Passeriformes	Pycnonotidae	Mountain bulbul	*Ixos mcclellandii*	-	LC	LC	0.02	2.33	1
19	Passeriformes	Pycnonotidae	Collared finchbill	*Spizixos semitorques*	-	LC	LC	0.02	2.33	1
20	Passeriformes	Phylloscopidae	Inornate warbler	*Phylloscopus inornatus*	-	LC	LC	0.01	2.33	2
21	Passeriformes	Scotocercidae	Manchurian bush warbler	*Horornis canturians*	-	LC	LC	0.01	2.33	1
22	Passeriformes	Scotocercidae	Rufous-face warbler	*Abroscopus albogularis*	-	LC	LC	0.01	2.33	2
23	Passeriformes	Paradoxornithidae	Vinox-throated parrotbill	*Sinosuthora webbiana*	-	LC	LC	0.01	2.33	1
24	Passeriformes	Timaliidae	Black-streak scimitar-babbler	*Erythrogenys gravivox*	-	LC	LC	0.06	9.30	10
25	Passeriformes	Timaliidae	Streak-breasted scimitar-babbler	*Pomatorhinus ruficollis*	-	LC	LC	0.03	9.30	5
26	Passeriformes	Alcippeidae	Huet’s fulvetta	*Alcippe hueti*	-	LC	LC	0.04	4.65	7
27	Passeriformes	Alcippeidae	David’s fulvetta	*Alcippe davidi*	-	LC	LC	0.06	13.95	9
28	Passeriformes	Leiothrichidae	White-throated laughingthrush	*Pterorhinus albogularis*	-	LC	LC	0.01	2.33	2
29	Passeriformes	Leiothrichidae	Buffy laughingthrush	*Pterorhinus berthemyi* *	II	LC	LC	0.01	2.33	1
30	Passeriformes	Leiothrichidae	White-browed laughingthrush	*Pterorhinus sannio*	-	LC	LC	0.02	6.98	2
31	Passeriformes	Leiothrichidae	Greater necklaced laughingthrush	*Pterorhinus pectoralis*	-	LC	LC	0.42	41.86	69
32	Passeriformes	Leiothrichidae	Red-billed leiothrix	*Leiothrix lutea*	II	LC	LC	0.15	13.95	24
33	Passeriformes	Leiothrichidae	Chinese hwamei	*Garrulax canorus*	II	NT	LC	0.23	16.28	38
34	Passeriformes	Turdidae	Pale thrush	*Turdus pallidus*	-	LC	LC	0.01	2.33	1
35	Passeriformes	Turdidae	White’s thrush	*Zoothera dauma*	-	LC	LC	0.22	20.93	36
36	Passeriformes	Turdidae	Grey-winged blackbird	*Turdus boulboul*	-	LC	LC	0.09	11.63	15
37	Passeriformes	Turdidae	Chinese blackbird	*Turdus mandarinus*	-	LC	LC	0.01	4.65	2
38	Passeriformes	Muscicapidae	Red-flanked bluetail	*Tarsiger cyanurus*	-	LC	LC	0.50	11.63	81
39	Passeriformes	Muscicapidae	Narcissus flycatcher	*Ficedula narcissina*	-	LC	LC	0.03	2.33	5
40	Passeriformes	Muscicapidae	Blue whistling-thrush	*Myophonus caeruleus*	-	LC	LC	2.31	30.23	377
41	Passeriformes	Turdidae	Mupin thrush	*Turdus mupinensis* *	-	NT	LC	0.01	2.33	1

Note: * indicates species endemic to China. National protection categories: I = Class I nationally protected wildlife; II = Class II nationally protected wildlife; - = not listed. China Vertebrate Red List and IUCN Red List categories: NE = Not Evaluated; LC = Least Concern; NT = Near Threatened; VU = Vulnerable. RAI = relative abundance index. SO, naive site occupancy, calculated as the percentage of camera-trap stations at which a species was detected at least once.

**Table 4:** Seasonal temporal overlap coefficients and peak-shift reliability of focal species between the cool-dry and warm-wet seasons.

Species	Cool-Dry Records	Warm-Wet Records	Δ_4_	95% CI	Cool-Dry Peak	Warm-Wet Peak	Peak Shift (h)	Reliable Peak Shift
*Myophonus caeruleus*	216	161	0.896	0.821–0.954	11:02	10:34	−0.47	No
*Melogale moschata*	319	87	0.884	0.805–0.949	20:06	21:05	0.99	No
*Chrysolophus pictus*	121	98	0.854	0.765–0.935	07:56	09:29	1.55	No
*Dremomys pyrrhomerus*	243	141	0.824	0.745–0.896	06:37	07:11	0.56	No
*Arctonyx collaris*	155	123	0.791	0.704–0.873	19:55	02:12	6.29	No
*Paguma larvata*	120	200	0.729	0.639–0.820	20:03	00:03	3.99	Yes
*Muntiacus reevesi*	101	180	0.642	0.548–0.736	22:30	18:02	−4.46	No

Note: Δ_4_ is the coefficient of overlap between cool-dry and warm-wet seasonal activity-density distributions. Peak shift is calculated as warm-wet peak time minus cool-dry peak time on a circular 24 h scale. A peak shift was considered reliable only when the 95% bootstrap confidence interval did not include zero. All estimates were based on 1000 bootstrap resamples.

## Data Availability

The data presented in this study are available from the corresponding author upon reasonable request.
